# Epicardial pacing wires, a five year, single centre experience

**DOI:** 10.1186/1749-8090-8-S1-O89

**Published:** 2013-09-11

**Authors:** J Abbas, K Toombs, A Zaidi, JA Hoschtitzky

**Affiliations:** 1 Salford Royal NHS Foundation Trust, Salford, UK; 2 Manchester Heart Center, Central Manchester Foundation Trust, Manchester, UK

## Background

Indications for epicardial pacemaker insertion in the adult population include; complex congenital cardiac defects, difficult venous access, tricuspid valve pathology and in patients undergoing concomitant cardiac surgical procedures. It still remains unclear as to the best type of lead used for this.

## Methods

Between February 2008 and October 2012, 103 epicardial pacing wires were implanted in 70 adult patients. Of these leads, 46 were included in the study. Information regarding the perioperative period and follow-up was analysed to determine the patient’s clinical course and the durability of the pacing wires.

## Results

On implantation the mean threshold of all the leads was 1.1+/-0.8V at a mean pulse width of 0.5+/-0.7ms. The mean impedance of the leads was 673.4+/-268.1 OH. There was no difference in the implant data between unipolar and bipolar or steroid eluting and non-steroid eluting leads. There was a significantly lower pacing threshold in the steroid eluting leads at latest follow up (p=0.031). Similarly the pacing thresholds were lower in the bipolar leads when compared to the unipolar leads (p=0.026). There was no difference in impedance.

## Conclusion

Overall the epicardial pacing leads demonstrate satisfactory pacing thresholds and impedance trends in the follow-up time of this study. Bipolar leads were more durable than unipolar leads and leads which eluted steroid were favourable than those which didn’t.

